# The Influence of an Acute Exercise Bout on Adolescents’ Stress Reactivity, Interference Control, and Brain Oxygenation Under Stress

**DOI:** 10.3389/fpsyg.2020.581965

**Published:** 2020-11-10

**Authors:** Manuel Mücke, Sebastian Ludyga, Flora Colledge, Uwe Pühse, Markus Gerber

**Affiliations:** Department of Sport, Exercise and Health, Sport Science Section, University of Basel, Basel, Switzerland

**Keywords:** executive function, inhibitory control, fNIRS, psychosocial stress, physical activity, TSST

## Abstract

**Background:**

High psychosocial stress can impair executive function in adolescents, whereas acute exercise has been reported to benefit this cognitive domain. The aim of this study was to investigate whether an acute bout of aerobic exercise improves the inhibitory aspect of executive function and the associated dorsolateral prefrontal cortex (DLPFC) oxygenation when under stress.

**Methods:**

Sixty male high school students aged 16–20 years performed a Stroop task (baseline condition) and were randomly assigned to an exercise group (30 min on ergometer at 70% of maximum heart rate) and a control group (30 min of reading). Subsequently, all participants underwent a modified Trier Social Stress Test, which included a Stroop task under enhanced stress. The Stroop tasks in both conditions were combined with functional near-infrared spectroscopy to record changes in DLPFC oxygenation in response to the tasks. Stress reactivity was measured with saliva samples (cortisol, alpha-amylase), heart rate monitoring, and anxiety scores.

**Results:**

All stress parameters indicated increases in response to the stressor (*p* < 0.001), with higher alpha-amylase [*t*(58) = −3.45, *p* = 0.001, *d* = 1.93] and anxiety [*t*(58) = −2.04, *p* = 0.046, *d* = 0.53] reactions in the control compared to the exercise group. Controlling for these two parameters, repeated measures analyses of covariance targeting changes in Stroop interference scores showed no main effect of stress [*F*(1,58) = 3.80, *p* = 0.056, ηp^2^ = 0.063] and no stress × group interaction [*F*(1,58) = 0.43, *p* = 0.517, ηp^2^ = 0.008]. Similarly, there was no main effect of stress [*F*(1,58) = 2.38, *p* = 0.128, ηp^2^ = 0.040] and no stress × group interaction [*F*(1,58) = 2.80, *p* = 0.100, ηp^2^ = 0.047] for DLPFC oxygenation.

**Conclusion:**

Our study confirms potentially health-enhancing effects of acute exercise on some of the physiological and psychological stress reactivity indicators. However, our data do not support the notion of an effect on interference control and DLPFC activation under stress.

## Introduction

The physiological response to acute stress is characterized by the activation of the hypothalamus-pituitary-adrenal (HPA) axis, which results in the release of cortisol by the adrenal cortex, and the autonomic nervous system (ANS), which increases the activity of its sympathetic division under stress and initiates a number of processes such as increased release of adrenaline and increase in heart rate (Pruessner et al., 2010). While there is a healthy midrange of stress reactivity that is considered adaptive and useful for coping with certain stressors (Boyce and Ellis, 2005), high stress reactivity can be problematic, as it contributes to allostatic load (McEwen, 1998) and is associated with health concerns. As a recent systematic review revealed, higher levels of stress reactivity are associated with negative long-term effects on health, and in particular with increased risk of cardiovascular disease and immune system dysfunction (Turner et al., 2020).

Studies have also shown that the brain is affected eminently by acute stress. Stress-related changes in architecture and function of the prefrontal cortex (PFC) in particular have been investigated, as it is involved in the regulation of the stress response, but also reacts sensitively to high stress exposure (McEwen and Gianaros, 2010). For instance, cortisol can cross the blood–brain barrier and bind to mineralocorticoid (MR) and glucocorticoid receptors (GR) in the PFC (Lupien et al., 2009), and stress-induced increases in catecholamine levels can indirectly impair PFC functioning as well (Arnsten, 2009). The PFC is considered the highest-evolved brain region, as its principal task is processing higher-order cognitive functions that enable thoughtful, rational and planned behavior (Pruessner et al., 2010; Diamond, 2013). As a part of this, executive functions refer to top-down mental processes requiring working memory, cognitive flexibility or inhibitory control (Diamond, 2013). During homeostasis, behavior is largely regulated through these top-down processes. However, under acute psychological stress, function of the PFC is impaired, and a shift takes place from thoughtful, time-consuming top-down to sensory-driven, rapid bottom-up regulatory processes (Arnsten, 2009). In support of this shift in regulation, meta-analytic findings have shown that behavioral performance in tasks requiring working memory, cognitive flexibility or interference control is impaired under acute stress (Shields et al., 2016).

Interference control, as an important subtype of inhibition, can be assessed with the Stroop color-word task. This task consists of two conditions, where color words are presented either in compatible or incompatible ink color, and requires participants to react to the ink color while ignoring the meaning of the written word. The time delay and/or the increased number of errors caused by the conflict in the incompatible condition is called the Stroop interference effect (Vanderhasselt et al., 2009). Neuroimaging studies suggest that among different brain regions, the dorsolateral prefrontal cortex (DLFPC) in particular is activated during Stroop tasks. This has been associated with the upregulation of the attentional set in order to process the stimulus interference on incompatible trials (Vanderhasselt et al., 2009). Additionally, in studies employing functional near-infrared spectroscopy (fNIRS), better Stroop performance (i.e., less interference) has been associated with the dominance of left-lateralized DLPFC activation (Zhang et al., 2014; Ludyga et al., 2019a).

As recent research has shown, adolescents are particularly at risk of experiencing negative effects of stress on cognition. According to the World Health Organization and national psychological health surveys (American Psychological Association, 2014; Güntzer, 2017; World Health Organization, 2019), adolescents have to cope with an increasing number of psychosocial stressors, while their physiological stress response mechanisms and psychological coping strategies are still developing. It is unsurprising that better stress coping strategies were the main health need reported by Swiss adolescents (Jeannin et al., 2005). Moreover, adolescents have been reported to have higher stress reactivity than other age groups (Romeo, 2010), and there are indications that adolescents might be particularly vulnerable to negative effects of stress on the prefrontal cortex (Lupien et al., 2009). This highlights the need for research on factors that can potentially mitigate negative effects of acute stress on executive functioning in this age group.

In this regard, the investigation of the effects of an acute exercise bout seems promising for a number of reasons. Firstly, moderate acute aerobic exercise has been found to elicit small-to-moderate improvements in inhibitory control and other executive functions (Ludyga et al., 2016). In adolescents, these temporary improvements appear to last at least 60 min after cessation of the exercise session (Ludyga et al., 2019b). Moreover, some studies suggest that acute exercise benefits interference control via increased oxygenation of the DLPFC. Using fNIRS, Ji et al. (2019) and Endo et al. (2013) showed that positive effects of acute exercise on Stroop performance were accompanied by changes in DLPFC oxygenation, and several studies reported that acute exercise at mild (Byun et al., 2014) or moderate intensity (Yanagisawa et al., 2010) evoked a predominantly left-lateralized activation of the DLPFC, also associated with improved Stroop performance. This suggests that acute exercise benefits interference control via a change toward a dominance of the left DLPFC. Secondly, researchers have suggested that exercise has stress-modulating properties. According to the Cross-Stressor-Adaptation Hypothesis, exercise causes stress-like reactions in the human body, and repeated exercise has been shown to cause a reduction of the stress response to exercise (habituation) (Hackney, 2006), which can potentially transfer to other stressors as well (Sothmann, 2006). Systematic reviews of the literature showed that study results on such transfer effects to psychosocial stress are still inconclusive (Jackson and Dishman, 2006; Mücke et al., 2018). However, cross-sectional studies (e.g., Rimmele et al., 2007), and a randomized controlled trial (Klaperski et al., 2014) using the Trier Social Stress Test (TSST), a psychosocial stressor task with high effectivity, reliability and ecological validity, showed attenuated stress reactivity of the HPA axis and the ANS in fitter participants and in those who participated in an exercise program, respectively. Moreover, initial evidence suggests that similar effects already occur after a single bout of aerobic exercise (Zschucke et al., 2015). Accordingly, acute exercise could mitigate potential negative effects of psychosocial stress on executive functioning via two different pathways—either by facilitating executive functioning, or by reducing the magnitude of the reaction to the stressor.

Therefore, the primary aim of the present study was to examine the effects of an acute bout of moderate aerobic exercise on interference control under the influence of psychosocial stress in male adolescents. Studies have found increased performance in interference control to be associated with more left-lateralized activation of the dorsolateral prefrontal cortex (Yanagisawa et al., 2010; Byun et al., 2014). Accordingly, it was hypothesized that compared to a control condition, acute exercise mitigates negative effects of stress on interference control, and is therefore associated with better behavioral interference control and more left-lateralized DLPFC activation than the control condition. As a secondary aim, the effects an acute bout of aerobic exercise on stress reactivity were investigated.

## Materials and Methods

### Participants

In total, 60 participants were recruited via advertisements, flyers and personal contact. Only male, healthy, right-handed (as verified with the Edinburgh Handedness Inventory, Oldfield, 1971) persons between 16 and 20 years of age were included. All participants were fluent German speakers. To standardize educational status, only participants currently attending academic high schools were admitted. Other studies showed that the level of regular physical activity can influence stress reactivity (Klaperski et al., 2014). Therefore, only participants who were not completely inactive, but who reported between two and six hours of exercise per week were included. Participants were informed about the study procedures at least 3 days prior to the data assessment and provided informed consent. All study procedures were in accordance with ethical principles of the Declaration of Helsinki and approval was obtained by the local ethics committee (Ethikkommission Nordwest- und Zentralschweiz, project number: 2018-01775) before the start of the study.

### Study Design

**FIGURE 1:**
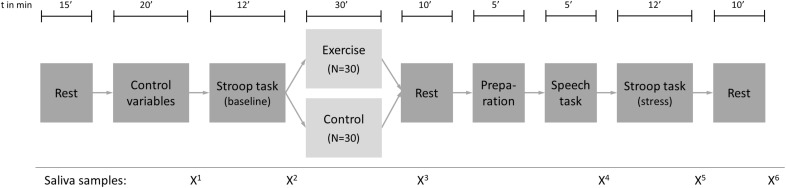
Study design.

The study design is depicted in Figure 1. Participants were randomly assigned to the exercise group (*N* = 30) or the control group (*N* = 30). The amount of self-reported regular physical activity was used as a stratum in order to create groups with similar physical activity behavior. As a cut-off, an amount of vigorous physical activity (VPA) of 180 min per week, as reported in the International Physical Activity Questionnaire (IPAQ), was used. This cut-off was chosen because it was the average weekly VPA in a previous study with a very similar sample (Mücke et al., 2020). All appointments were scheduled in the afternoon at either 13:00 or 16:00 to minimize the potential impact of variations in diurnal cortisol levels (Kudielka et al., 2004). Upon arrival, participants rested for 15 min to reduce the influence of possible stress factors before and/or during arrival. Body height and weight were then measured objectively with a stadiometer and an electronic scale (Tanita BC-601, Tokyo, Japan), respectively, and participants filled in a questionnaire including age (in years), socio-economic status (one item), physical activity [International Physical Activity Questionnaire (IPAQ); Craig et al., 2003], sleep complaints [7-item Insomnia Severity Index (ISI); Gerber et al., 2016], chronic stress [10-item Perceived Stress Scale (PSS); Klein et al., 2016], mental toughness [18-item short form of the Mental Toughness Questionnaire (MTQ18); Gerber et al., 2018], and psychopathology [25-item Strengths and Difficulties Questionnaire (SDQ); Goodman, 2001]. The validity of all psychological instruments has been established previously and all measures showed acceptable internal consistency in the present sample (Cronbach’s alpha > 0.67 for all psychometric variables). Subsequently, an fNIRS head cap (NIRSport, NIRx Medical Technologies, Berlin, Germany) was fitted to the participants’ head, sensors were calibrated and a Stroop Color-Word task was performed (these processes are described in detail in Section “Interference Control and Prefrontal Brain Activity”). During the next 30 min, the control group read an article from a magazine of their choice, while the exercise group performed an exercise session at moderate intensity on a bicycle ergometer (R60, Vision Fitness, Frechen, Germany). After the intervention, the head cap was mounted again. Subsequently, a modified version of the Trier Social Stress Test (TSST) was performed as described in Section “Stress Paradigm and Measurement of Stress Reactivity”. The time delay between the end of the exercise or control condition and the beginning of the stress task was approximately 10 min. Within the TSST setup, the Stroop Color-Word task was performed again, with the difference that this time participants were instructed in a way that contributed to an increase in psychosocial stress (see Section “Stress Paradigm and Measurement of Stress Reactivity”). The appointment ended with a 10 min resting period, and all participants received a financial compensation of 70 CHF for their participation. Before and after the Stroop tasks, the intervention (acute exercise vs. reading) and the stress test, and after the resting period, saliva samples were collected with Salivette Blue Cap (Sarstedt, Nümbrecht, Germany) to control for saliva cortisol and alpha-amylase levels (see Figure 1 and Section “Stress Paradigm and Measurement of Stress Reactivity”).

### Exercise Session

During the exercise session, participants pedaled at a constant speed (70–80 rpm). Moderate intensity was defined as 70% of maximum heart rate (HR_*max*_), which was calculated with the formula HR_*max*_ = 208 − 0.7 × age (Tanaka et al., 2001). Pedaling resistance was continuously adjusted according to the measured heart rate. Furthermore, subjectively perceived intensity was monitored every 5 minutes using rating of perceived exertion (Borg, 1982).

### Interference Control and Prefrontal Brain Activity

A computer-based version of the Stroop Color-Word task was used to assess interference control (Homack and Riccio, 2004). It consisted of compatible and incompatible trials. In compatible trials, color words appeared in the same ink color (e.g., “blue” printed in blue), whereas in incompatible trials, color words appeared in a different color of ink (e.g., “yellow” printed in green). To ensure similar visual content, the German color words “grün” (green), “gelb” (yellow), “blau” (blue), and “pink” were used. Participants were instructed to press a button corresponding to the color of ink, ignoring the actual meaning of the word, and to react as quickly and accurately as possible. Stimuli were presented for 250 ms, and responses were collected within a 1250 ms time window. The inter-stimulus time varied randomly between 300 and 500 ms. The task included twenty test blocks, each lasting 22-24 s. The duration of the resting periods between the test blocks varied randomly between 10 and 15 s. Compatible and incompatible test blocks alternated and within each block, the stimuli appeared with equal probability and followed a fully randomized order. Before testing, two practice blocks were conducted for familiarization and to reduce learning effects. Illustrations of the Stroop task sequence and block design are presented in the Supplementary Material (Supplements 1, 2).

For analysis, an interference score was calculated as the difference between reaction time on incompatible trials minus reaction time on compatible trials. Only response-correct trials with reaction times ≥120 ms were used for calculation as shorter response times would be highly likely to indicate guesswork (Zhang et al., 2014). A lower interference score equals higher interference control. To check whether potential group differences were influenced by speed-accuracy trade-offs, response accuracy was recorded as well.

**FIGURE 2:**
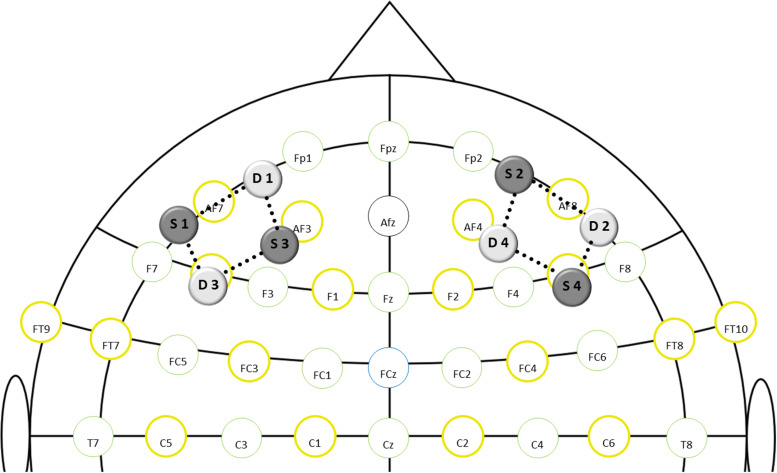
fNIRS montage layout in relation to standard EEG positions (S-source; D-detector; dotted lines-channels).

For measurement of DLPFC brain oxygenation during the Stroop task, a dual-wavelength (760 and 850 nm) continuous-wave fNIRS system with a sampling rate of 7.8125 Hz (NIRSport, NIRx Medical Technologies, Berlin, Germany) and the recording software NIRStar 15.2 (NIRx Medical Technologies, Berlin, Germany) were used. Eight optodes (4 illumination sources, 4 light detectors) were mounted into a flexible cap, which was then placed on the participant’s head. Optodes were equally distributed over the left and right DLPFC as shown in Figure 2. The DLPFC location was defined as described by Carlén (2017), and international 10:10 EEG positions were used as referencing points [for exact probe positions, see Supplementary Material (Supplement 4)]. The same montage has been used previously by Ludyga et al. (2019a). Spacers were used to keep the inter-optode distance constant at 3cm, which is considered the best compromise between high light penetration depth and sufficient signal-to-noise ratio (Ferrari and Quaresima, 2012; Tak and Ye, 2014). A black overcap was used to minimize the impact of ambient light. Additionally, the surrounding noise was reduced to a minimum and participants were instructed to avoid head movements and speaking during the Stroop task. Recording procedures were in line with existing quality standards (Orihuela-Espina et al., 2010) and recommendations for fNIRS assessments in exercise-cognition research (Herold et al., 2018).

After recording, fNIRS data was processed with Homer2 version 2.3 (Huppert et al., 2009). The processing stream followed the one proposed by Brigadoi et al. (2014) and is described in detail in Ludyga et al. (2019a). Artifacts exceeding defined thresholds were automatically marked and manually verified. Based on the results of systematic comparisons of artifact correction techniques (Scholkmann et al., 2010; Cooper et al., 2012), spline interpolation was used to correct marked artifacts, followed by a frequency filter with a low cut-off at 0.01 Hz (Yennu et al., 2016) and a high cut-off at 0.5 Hz (Brigadoi et al., 2014). Block averages were created for compatible and incompatible test blocks with the 2 s period preceding the test block used as reference. For the calculation of left and right DLPFC oxygenation, the average of all 4 channels on each side was calculated because test-retest reliability has been found to be higher at cluster level compared to individual channels (Schecklmann et al., 2008). Oxygenation related to Stroop interference was calculated as average oxygenation during incompatible minus compatible test blocks (Δ_*OXY*_).

### Stress Paradigm and Measurement of Stress Reactivity

Psychosocial stress was induced using a modified version of the TSST (Kirschbaum et al., 1993). It consisted of an anticipation phase and a mock job interview, followed by a Stroop task with adapted instructions designed to enhance psychological stress. Both the mental arithmetic task used in the original TSST, as well as the Stroop task implemented in our modified version, have been used as cognitive stressors in previous studies (Dickerson and Kemeny, 2004). In our psychosocial stressor, two motivated performance tasks (speech and cognitive test) were combined with the additional element of uncontrollability and socio-evaluative threat. This combination has been shown to be more effective in triggering a physiological stress response than other laboratory stressors consisting only of a single task (Dickerson and Kemeny, 2004). The following protocol was used: after a 5 min preparation phase, participants performed a 5 min unrehearsed speech in front of a committee of two (one male and one female), followed by a 10 min Stroop task. Participants were instructed to imagine a situation in the near future when they finished school, were looking for a job and were offered an interview for their dream job. The committee was introduced to the participants as the manager of the company and an assistant who is specialized in the interpretation of body language and voice frequency. Throughout the speech, the committee showed neutral facial expressions and only used standardized responses (e.g., “You still have time left. Please continue.”). Subsequently, the Stroop task was performed as described in the section above, with the following additions. The committee informed the participant that his test performance was visible on their screen and that they were able to compare his performance directly to other participants’ data. The committee further remarked that if he did not perform well, he would not get the job, and the financial compensation for study participation would be reduced.

Stress reactivity was measured using saliva samples (for analysis of cortisol and alpha-amylase concentrations), heart rate monitoring and self-reported state-anxiety scores. While salivary free cortisol represents the reactivity of the HPA axis (Kudielka et al., 2004), salivary alpha-amylase is known to be reflective of the stress response of the autonomic (more specifically: sympathetic) nervous system (Nater and Rohleder, 2009). Saliva samples were collected at several time points during the appointment as shown in Figure 1. After data assessment, they were first stored at −20°C and then sent to the Biochemical Laboratory of the University of Trier, Germany, for analysis of cortisol (in nmol/l) and alpha-amylase (in U/ml) concentrations using time-resolved fluorescence immunoassay. As a parameter indicating the activation of the sympathetic nervous system in reaction to stress, heart rate was monitored continuously throughout the stress test. For the purpose of data analysis, 1 min intervals were averaged. Baseline heart rate was measured for 2 min before introduction of the stress test. Psychological stress reactions were measured before and after the stressor using 5 items of the state-anxiety scale of the State-Trait Anxiety Inventory (STAI; Laux et al., 1981; Cronbach’s alpha = 0.72). After recoding inverted items, a sum score was calculated. It ranges from 5 to 20, with higher scores indicating higher anxiety.

### Statistical Analysis

A power analysis was calculated with G^∗^Power software. As no data on the effects of acute exercise on interference control under stress exists, yet, our power analysis was based on a meta-analysis by Verburgh et al. (2014), who reported moderate effects of acute exercise on interference control in adolescents. It resulted in a minimum number of 52 participants (parameters: repeated measures ANOVA, within-between interaction; effect size f = 0.20; alpha error probability = 0.05; power = 0.80; number of groups: 2; number of measurements: 2; correlation among repeated measures = 0.50; non-sphericity correction = 1).

Following Pruessner et al. (2003), for physiological stress reactivity (cortisol, alpha-amylase and heart rate reactivity), the area under the curve with respect to the increase (AUC_*I*_) was calculated. Since alpha-amylase shows an immediate increase after stimulation of the ANS (Nater and Rohleder, 2009), samples 3–6 (Figure 1) were used. Salivary cortisol levels usually rise with about 10 min delay relative to stressor onset (Foley and Kirschbaum, 2010). Therefore, samples 4–6 were used to assess cortisol reactivity. For heart rate reactivity, the 2 min before the introduction of the TSST were averaged and used as a baseline, and the AUC_*I*_ was calculated from the subsequent averaged 1 min intervals until stressor cessation. Psychological stress reactivity was defined as the difference of the post-stress minus pre-stress anxiety score. Subsequently, potential group differences in baseline values and stress reactivity (AUC_*I*_) were analyzed using separate independent *T*-tests.

The effect of exercise (compared to the control condition) on interference control under stress was examined using a repeated-measures analysis of variance (rANOVA) with stress (baseline Stroop interference vs. Stroop interference under stress) as within-subject variable and group (exercise vs. control) as between-subjects factor. In a second run of the analysis, stress reactivity parameters that showed group differences were added as covariates.

The effect of exercise on DLPFC oxygenation under stress was investigated using a rANCOVA with stress (Δ_*OXY*_ at baseline vs. Δ_*OXY*_ under stress) and hemisphere (Δ_*OXY*_ left vs. Δ_*OXY*_ right DLPFC) as within-subject variables and group (exercise vs. control) as between-subject factors. Heart rate during the Stroop task was added as a covariate, because fNIRS data can potentially be affected by systemic changes (Herold et al., 2018). For all rAN(C)OVA, main effects and interactions were reported. Effect sizes were classified as small (*d* ≥ 0.2; ηp^2^ ≥ 0.01), medium (*d* ≥ 0.5; ηp^2^ ≥ 0.06), or large (*d* ≥ 0.8; ηp^2^ ≥ 0.14) (Cohen, 1988). An alpha level of *p* ≤ 0.05 was considered statistically significant. All statistical analyses were performed with SPSS 26 (IBM Corporation, Armonk, NY, United States).

## Results

### Sample Characteristics and Exercise Session

Characteristics of the sample are presented in Table 1. The exercise and control groups did not differ significantly in any of the anthropometric, sociodemographic or psychological control variables. During the exercise session, the average (standard deviation) heart rate and rating of perceived exhaustion were 128.5 (7.9) beats per minute and 14.0 (1.0), respectively. Average heart rate during the exercise session was significantly higher compared to the control condition [69.8 (10.0) beats per minute; *t* = 24.9, *p* = 0.000, *d* = 6.60] and represented 65.7 (4.0)% of HR_*max*_.

**TABLE 1:** Comparison of group characteristics (independent *T*-test).

	Exercise group M ± SD	Control group M ± SD	*p*
Age in years	17.9 ± 1.2	17.9 ± 1.3	0.999
BMI in kg/m^2^	22.9 ± 3.1	22.8 ± 3.2	0.944
Socioeconomic status	3.3 ± 0.6	3.2 ± 0.6	0.667
MVPA in min/week (IPAQ)	308.3 ± 237.4	288.7 ± 157.8	0.707
Chronic stress (PSS)	13.9 ± 4.5	15.0 ± 5.3	0.365
Mental toughness (MTQ18)	45.6 ± 7.3	46.2 ± 6.8	0.730
Psychopathology (SDQ)	9.23 ± 4.1	9.23 ± 4.4	0.999
Insomnia (ISI)	7.6 ± 5.0	6.3 ± 4.1	0.304

*BMI, body mass index; IPAQ, International Physical Activity Questionnaire; ISI, Insomnia Severity Index; M, mean; MTQ18, Mental Toughness Questionnaire; MVPA, Moderate-to-vigorous physical activity; PSS, Perceived Stress Scale; SD, standard deviation; SDQ, Strengths and Difficulties Questionnaire.*

### Stress Reactivity

To enable the investigation of interference control under stress, our study design required differences in stress parameters between both Stroop task conditions (baseline and under-stress). As a manipulation check, paired *T*-tests were calculated. All physiological stress parameters indicated higher stress during the Stroop task performed under stress compared to the baseline condition (for cortisol and alpha-amylase directly after both Stroop tasks: *p* < 0.001; for heart rate during both Stroop tasks: *p* = 0.03). When using the measurement points directly after each Stroop task, self-reported psychological stress did not differ between both conditions (*p* = 0.80). However, between both measurement points, the stress test did evoke a measurable psychological stress response (see below).

**FIGURE 3:**
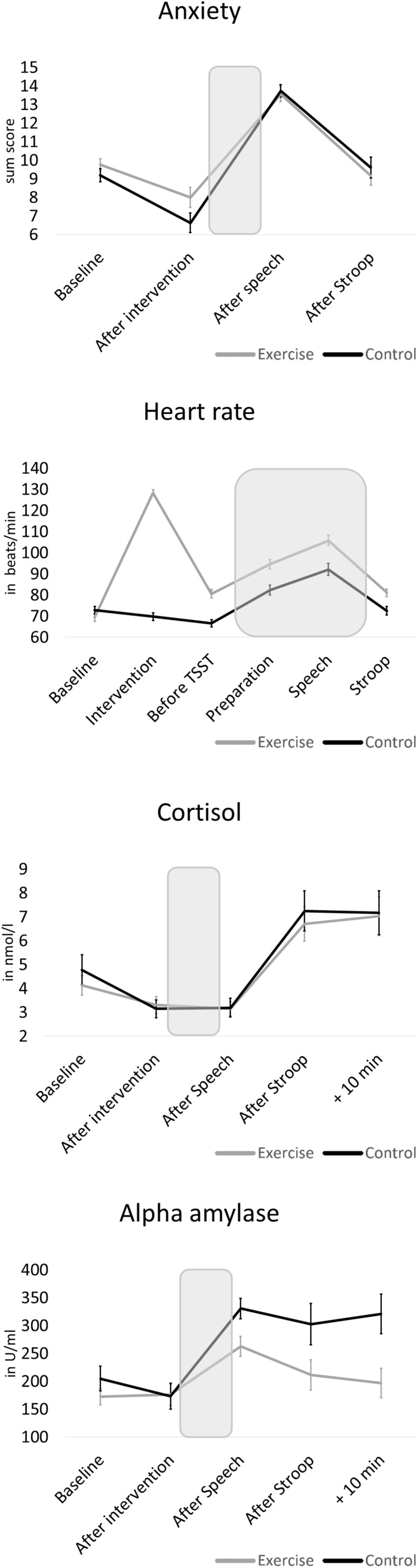
Mean physiological and psychological stress reactivity of the exercise group and the control group. The shaded areas indicate the stressor (preparation and speech task). Error bars are standard errors of the mean (SEM).

Changes in physiological and psychological stress parameters in response to the modified TSST are depicted in detail in Figure 3. Comparing both groups, independent *T*-tests revealed no baseline difference (that is: after exercise or control intervention, before stress test) for cortisol (*t* = −0.07, *p* = 0.943, *d* = 0.02) and alpha-amylase (*t* = 0.11, *p* = 0.914, *d* = 0.03). However, the control group showed significantly less anxiety (*t* = 2.55, *p* = 0.014, *d* = 0.67) and lower heart rate (*t* = 5.57, *p* < 0.001, *d* = 1.46) before the stress task than the exercise group. With regard to stress reactivity, we found a significant increase across the total sample in all four parameters (*p* < 0.001). However, groups differed in stress responses of alpha-amylase [*t*(58) = −3.45, *p* < 0.001, *d* = 1.93] and anxiety [*t*(58) = −2.04, *p* = 0.046, *d* = 0.53], with large and medium effect sizes, respectively, indicating higher stress reactivity in the control group. No differences between the exercise and control groups were present for cortisol [*t*(58) = −0.43, *p* = 0.668, *d* = 0.11] and heart rate reactivity [*t*(57) = -0.48, *p* = 0.636, *d* = 0.13].

### Inhibitory Performance

**FIGURE 4:**
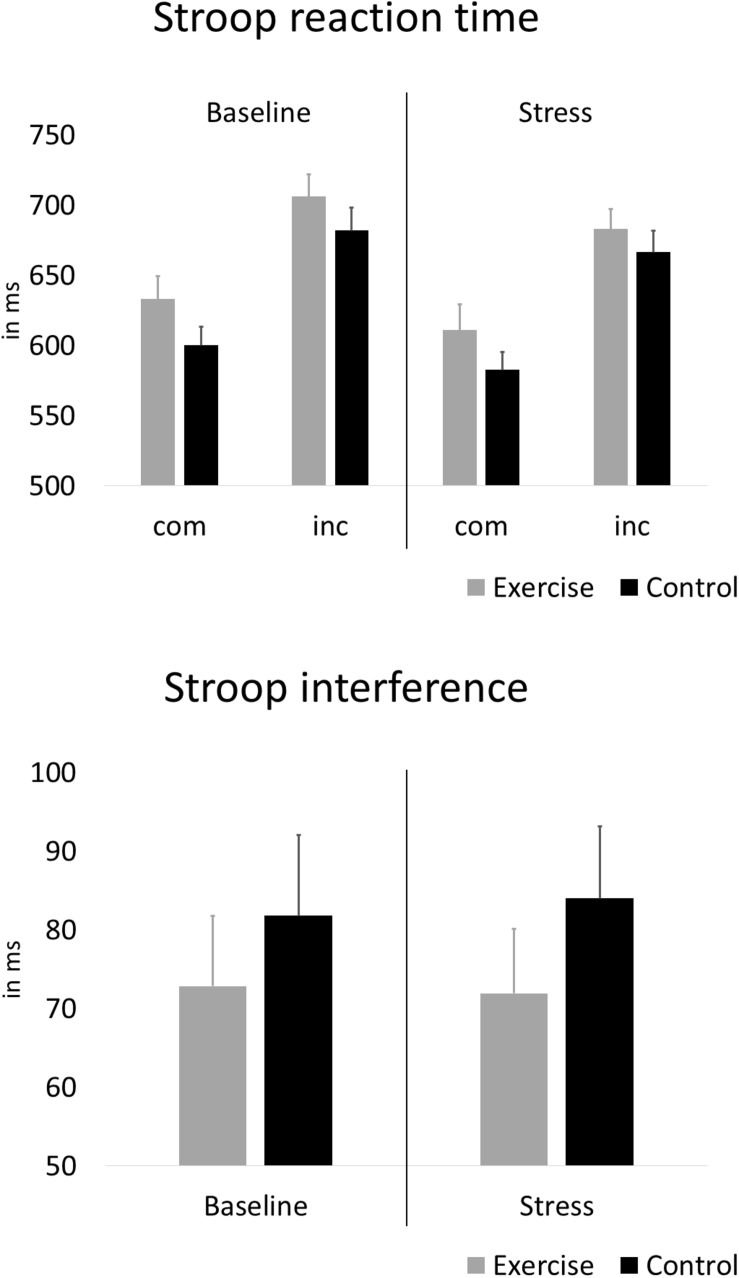
Average Stroop reaction time during compatible (com) and incompatible (inc) test blocks and interference scores before and under stress. Error bars are standard errors of the mean (SEM).

Figure 4 depicts the reaction times and interference scores for both groups during the baseline Stroop task and the Stroop task under stress. With regard to effects of exercise, and stress, on interference scores, the rANOVA showed no statistically significant main effect of stress [*F*(1,58) = 0.01, *p* = 0.925, ηp^2^ = 0.000] and no stress × group interaction [*F*(1,58) = 0.05, *p* = 0.826, ηp^2^ = 0.001]. After further including alpha-amylase and psychological stress reactivity, the rANCOVA again showed no statistically significant main effect of stress [*F*(1,58) = 3.80, *p* = 0.056, ηp^2^ = 0.063] and no stress × group interaction [*F*(1,58) = 0.43, *p* = 0.517, ηp^2^ = 0.008].

Furthermore, response accuracy interference was analyzed to control for potential speed-accuracy trade-offs. Repeating the same analyses with response accuracy revealed no statistically significant main effect of stress [*F*(1,58) = 1.826, *p* = 0.182, ηp^2^ = 0.031] and no stress × group interaction [*F*(1,58) = 3.79, *p* = 0.056, ηp^2^ = 0.061]. However, at baseline the exercise group showed lower response accuracy during incompatible trials compared to the control group [*T*(1,58) = −2.65, *p* = 0.010, *d* = 0.70]. Response accuracy scores of both groups are presented in the Supplementary Material (Supplement 3).

### DLPFC Oxygenation

**FIGURE 5:**
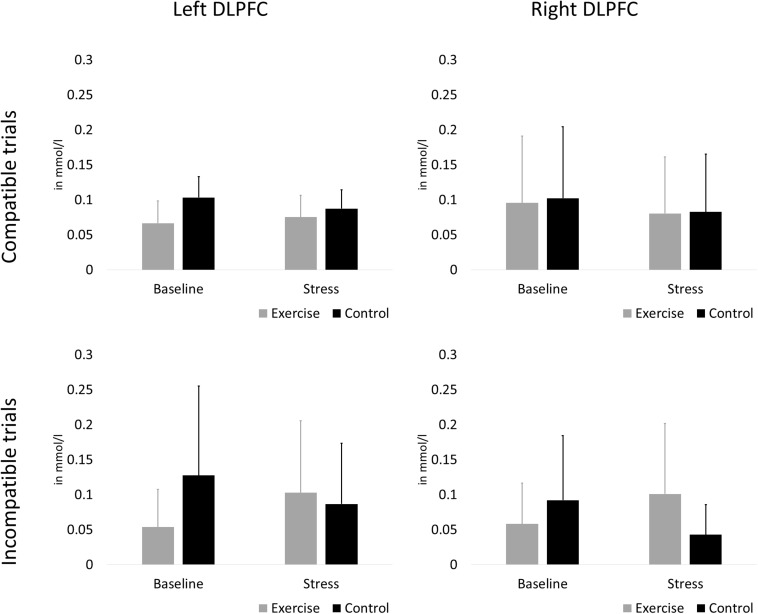
Oxygenation changes in the left **(A,C)** and right **(B,D)**. DLPFC during compatible **(A,B)** and incompatible **(C,D)** test blocks. Error bars are standard errors of the mean (SEM).

The rANCOVA showed no statistically significant main effect of stress [*F*(1,58) = 2.38, *p* = 0.128, ηp^2^ = 0.040], no stress × group interaction [*F*(1,58) = 2.80, *p* = 0.100, ηp^2^ = 0.047], and no hemisphere × group interaction [*F*(1,58) = 0.76, *p* = 0.387, ηp^2^ = 0.013]. All other main effects and interaction terms did not reach statistical significance (*p* < 0.601). Oxygenation changes in the left and right DLPFC during compatible and incompatible test blocks are presented in Figure 5.

## Discussion

This study aimed to investigate the effect of an acute exercise bout on interference control under stress and corresponding oxygenation differences in the left and right DLPFC. In our study, we found no indication of differences between the exercise group and control group with regard to interference control under stress, and controlling for differences in stress reactivity did not change this result. Corresponding oxygenation differences in left and right DLPFC also did not differ between groups. While the stress test elicited significant reactions in all stress reactivity parameters and across both groups, we found higher alpha-amylase reactivity and higher increases in anxiety in the control group compared to the exercise group.

### Acute Exercise and Interference Control Under Stress

A wealth of studies have already investigated the effects of acute exercise on executive functioning without enhanced stress in various age groups. Systematic reviews and meta-analyses consistently reported small but significant effects, and demonstrated that acute exercise is beneficial for subsequent executive functioning across all age groups (Tomporowski, 2003; Chang et al., 2012; Ludyga et al., 2016), although age groups that are typically characterized by developmental changes seem to benefit more than others (Guiney and Machado, 2013; Ludyga et al., 2016). In a meta-analysis compiling data on preadolescent children (6-12 years), adolescents (13-18 years) and young adults (18-35 years), Verburgh et al. (2014) reported moderate effects of acute exercise on inhibition/interference control in children and adolescents, and small-to-moderate effects in young adults. More recent empirical findings on adolescents corroborated this pattern for interference control (Browne et al., 2016; Peruyero et al., 2017; Park and Etnier, 2019). However, no studies so far looked into the effects of acute exercise on interference control under the influence of psychosocial stress. Previous studies reported negative effects of acute stress on executive functions, including interference control (Shields et al., 2016). As maintaining high executive functioning under stress is of great importance for success in education and professional life, and higher executive functioning under stress has been shown to be associated with better health (Williams and Thayer, 2009; Shields et al., 2017), research on mitigating factors is important.

In this study, we present initial insights into the influence of acute exercise on interference control in the presence of acute psychosocial stress. Despite the promising effects on interference control in situations without additional stress, which previous studies reported to be most pronounced in young people, our study with participants in later stages of adolescence did not show such effects in the presence of acute psychosocial stress. However, these results, which refer to interference scores based on reaction time, can be influenced by differences in response accuracy. In our study, during incompatible trials the exercise group showed worse response accuracy at baseline, but not under stress, and a medium effect size (non-significant, however) pointed toward a stress × group interaction on accuracy interference, indicating potential group differences in response accuracy in favor of the exercise group. These potential group differences in response accuracy might indicate a speed-accuracy trade-off and might have caused effects on the main outcome to disappear. Nevertheless, compared to the results other studies reported for stress-free conditions, exercise effects on interference control appeared to be smaller or absent under stress, and based on our data, we cannot generally recommend acute exercise to enhance interference control under stress. Individuals differ largely in how they perceive and react to stress, and researchers argue that the vulnerability to, and resilience against potential negative effects of acute stress on cognition might vary largely among individuals (Sandi, 2013). While we took the most important anthropometric, sociodemographic and psychological confounders into account, it cannot be ruled out that among other individual factors, the effects of acute exercise were too small to be detected. Our exercise intervention comprised 30 min of ergometer exercise at a constant, moderate intensity (on average 66% of HR_*max*_). While interventions of similar type, duration and intensity proved to be effective in enhancing interference control (Alves et al., 2012; Chang et al., 2015), it is possible that under acute stress, different exercise modalities might have yielded more favorable results. For instance, meta-analytical findings by Gu et al. (2019) indicate that open-skill exercise might be more effective for improving cognitive functioning than closed-skill exercise, and Ludyga et al. (2018) showed beneficial effects if aerobic and coordinative demands are combined. On the other hand, ergometer cycling seems to have superior effects on cognitive performance compared to treadmill running exercise (Lambourne and Tomporowski, 2010), and researchers found similar effects for aerobic and strength (Alves et al., 2012) or coordinative exercise (Ludyga et al., 2017) on inhibitory control. To elicit improvements in executive functioning, exercise durations between 20 and 60 min are deemed optimal (Tomporowski, 2003; Lambourne and Tomporowski, 2010). With regard to exercise intensity, studies reported beneficial effects on Stroop performance following low and high (Peruyero et al., 2017), and moderate intensity exercise (Browne et al., 2016; Park and Etnier, 2019). Studies investigating a dose-response relationship suggested an inverted-U-shaped effect, with best results for moderate exercise (McMorris and Hale, 2012). It is noteworthy that depending on intensity, exercise itself can have an impact on stress parameters. According to Hackney (2006), exercise that surpasses an intensity of 50-60% of the maximal oxygen uptake (VO_2*max*_) increases circulating concentrations of cortisol. In our study, stress parameters did not rise in response to the exercise session (see Figure 3), which means that they might not have surpassed this VO_2_ threshold. This might have had an influence on our results, and future studies should look into the effect of exercise intensity and exercise-induced stress on executive functions. Overall, the findings listed above apply to effects of different exercise modalities on executive functioning without the additional element of psychosocial stress, and future studies are encouraged to investigate whether different exercise modalities have distinct effects on executive functioning under stress.

The absence of the hypothesized beneficial effect of acute exercise in our study might in part be explained by the absence of the expected negative impact of stress on interference control. Our results showed no main effect of stress on the interference score, indicating that in our study, the stressor did not change interference control in the overall sample. This was surprising, because other studies reported impaired inhibitory performance under stress (Sanger et al., 2014; Roos et al., 2017), and meta-analytical findings, although based on a small number of studies, suggested that the negative effect of acute stress on interference control is independent of stress severity and stress type (Shields et al., 2016). As our stress reactivity analysis revealed, the stressor elicited significant increases in all measured physiological and psychological indices of stress reactivity. Nevertheless, participants’ ratings of anxiety after the baseline Stroop task, and the Stroop task under stress, did not differ significantly (cp. Figure 3). Studies showed that impairments in Stroop performance under stress can largely be attributed to subjective stress perceptions (Henderson et al., 2012). However, other studies also found associations of HPA axis and ANS reactivity with impaired inhibitory control (Sanger et al., 2014; Roos et al., 2017). As our study did not include a control condition without stress, we were not able to fully control for the influence of potential practice effects on the results. Participants might have performed better under stress because an assessment of inhibitory control without stress took place beforehand (see limitations). In conclusion, it remains unclear why the stressor failed to elicit the expected decline in behavioral interference control, and more studies on the effect of stress on executive functioning, and on the potential role of exercise, are necessary.

### Associations With DLPFC Oxygenation

Along with behavioral parameters, DLPFC oxygenation was measured to account for neurophysiological mechanisms underlying interference control. Recent fNIRS studies demonstrated that more left-lateralized DLPFC oxygenation was associated with higher interference control (Zhang et al., 2014; Ludyga et al., 2019a). This effect has been attributed to differences in left and right DLPFC activation when stimulus conflict is anticipated and up-regulation of the attentional set is required (Vanderhasselt et al., 2009). According to lateralized Stroop studies, interference effects might be greater in the left hemisphere because, compared to the right hemisphere, the left hemisphere presents an overall advantage on most verbal tasks (Belanger and Cimino, 2002). Moreover, research with fNIRS showed that positive effects of exercise on interference control might be mediated by DLPFC lateralization. In 25 young adults, Byun et al. (2014) observed improved performance in a Stroop color-word matching task after a 10min bout of mild ergometer exercise, which was accompanied by pronounced activation of the left DLPFC in relation to Stroop interference. In a sample of 60 older adults, Hyodo et al. (2016) reported correlations between higher aerobic fitness and better Stroop performance, and mediation analysis revealed that this relationship was mediated by more left-lateralized DLPFC activation. In a recent study utilizing a combined fNIRS-EEG approach, our research group investigated mechanisms underlying the association between aerobic fitness and interference control in a sample similar to the present study (Ludyga et al., 2019a). While both left-lateralized DLPFC oxygenation, and greater N450 negativity, were associated with better Stroop performance, only N450 negativity mediated the fitness-interference control relationship. Again, no studies are available that investigated associations between exercise and interference control in the presence of acute psychosocial stress, and the present study provides first insights into this relationship. Overall, our data indicate a tendency toward left-lateralized activation in both groups and in both conditions (cp. Figure 5). No systematic differences in DLPFC oxygenation occurred between both groups and conditions. These results match our findings with regard to behavioral interference control, but provide no support for our hypothesis of increased left-lateralized DLPFC activity in the exercise group. From other studies we know that exercise improves interference control via facilitation of DLPFC activation (e.g. Yanagisawa et al., 2010; Byun et al., 2014), and that acute stress affects the PFC (Arnsten, 2009). While our study only assessed stress effects on activation and functioning of the DLPFC, our results do not allow conclusions on the activation of other PFC regions under stress, and potential corresponding effects of acute exercise.

### Exercise Effects on Stress Reactivity

In our study, we observed that the acute exercise group showed lower stress reactivity than the control group in the parameters alpha-amylase and anxiety, but not in the parameters cortisol and heart rate. While these group differences in stress reactivity were not related to significant changes in interference control, they are relevant for different reasons. As research shows, the phase of adolescence, compared to other age groups, is characterized by a typical increase in stress reactivity in response to acute psychosocial stressors (Lupien et al., 2009; Stroud et al., 2009). The combination of frequent stress exposure in this age group (American Psychological Association, 2014) and potentially high stress reactivity, increases the risk of corresponding future stress-related health issues (Redmond et al., 2013; Turner et al., 2020). Therefore, a reduction in stress reactivity in the face of psychosocial stressors is often desirable. Our results now show that acute exercise has such potentially health-beneficial effects on stress reactivity.

Changes in stress reactivity in relation to exercise have been observed before, and are often explained with habituation effects of the stress response systems when exposed to regular exercise (Herman et al., 2005; Hackney, 2006), which then transfer to the reaction to psychosocial stressors (Sothmann, 2006). While this has often been demonstrated for regular exercise (Mücke et al., 2018), only few studies investigated such effects after a single exercise bout. Three relatively recent studies investigated the effects of acute exercise on physiological stress reactivity in young adults (Zschucke et al., 2015; Wood et al., 2018; Wunsch et al., 2019). Interestingly, although these studies differed largely with regard to exercise type (walking vs. bicycle ergometer vs. treadmill), exercise intensity (moderate walking vs. 70% of their individual maximum load vs. 60—70% of maximum oxygen uptake), time delay from exercise to stressor (30 min vs. 10 min vs. 90 min delay), stress task (TSST-G vs. Montreal Imaging Stress Task), and control task (passive control vs. light stretching), they consistently reported attenuated cortisol and/or alpha-amylase reactivity in the exercise group, compared to the control group. This initial data demonstrates that the effects of acute exercise on stress reactivity seem to be fairly robust and are related to a wide range of exercise modalities. In our slightly younger sample of male adolescents, and with exercise parameters within the range of these previous studies, we show similar results with regard to alpha-amylase, which represents stress reactions of the autonomic nervous system (Nater and Rohleder, 2009). However, no such effects were observed with regard to cortisol. Different effects of the exercise session on these parameters are unlikely to be the explanation for this result, as directly after the exercise or control condition, alpha-amylase as well as cortisol levels did not differ between groups. Studies have already shown that the reactions of HPA axis and ANS system to psychosocial stressors can be dissociated (Schommer et al., 2003). However, in this particular case, the reasons for these differences remain unclear. Lastly, our study indicated transient effects of exercise on self-reported anxiety. In response to the stressor, we observed lower increases in anxiety in the exercise group, compared to the control group. After the stressor, both groups reported similar anxiety levels. As other studies so far focused on physiological stress parameters, there is a lack of research on acute exercise effects on psychological stress reactivity, and our findings provide initial support for improved coping with stressors that are characterized by uncontrollability and socio-evaluative threat after an acute bout of exercise. Further studies are necessary to confirm these initial results.

### Limitations

The results of our randomized, controlled examination have some limitations that need to be considered. As our sample consisted of healthy, male, right-handed adolescents with a rather high educational status, conclusions on other target groups need to be treated with caution. Further research with female participants, different age groups and educational status, or with clinical samples is necessary and could lead to different results. Furthermore, it is possible that different exercise conditions might have changed the results. It is noteworthy that in our study, a modified version of the TSST was used. The mental arithmetic task, as described in the original version by Kirschbaum et al. (1993), was replaced by a Stroop task in order to measure participants’ interference control under the direct influence of the psychosocial stressor. Although both mental arithmetic and Stroop tasks have been used as stressors before (Dickerson and Kemeny, 2004), and substantial differences in stress reactivity are therefore unlikely, direct comparisons of our results with other TSST studies are limited. In our study, an acute exercise group was compared to an active control group. However, both groups underwent the complete stressor task, and no “no-stress” control group was present. Therefore, our study did not control for the effects of repeated Stroop task exposure, and our results may be confounded by practice effects. However, other studies reported no such effects after repeated Stroop task administration (Browne et al., 2016), and since it would have affected both groups equally, a change of the general patterns of results because of practice effects is unlikely. Finally, despite its advantages in the assessment of cortical brain activity (Zhang et al., 2014), the use of fNIRS has some limitations. It has been shown that fNIRS measurements can be partially affected by skin blood flow and systemic effects (Tachtsidis and Scholkmann, 2016). However, we expect the effect of such artifacts to be small in our analyses, because all Stroop tasks in our study were conducted under standardized conditions (the participants were instructed to remain seated, to avoid speaking and to breathe regularly throughout the measurement to keep these parameters constant). Moreover, because we calculated Stroop interference related to DLPFC activation as the difference between incompatible and compatible trials, the shared potential global artifacts of both trial types should cancel each other out (Hyodo et al., 2016).

## Conclusion

Adolescents performing an acute exercise bout appear to show lower stress reactivity of the autonomic nervous system, and a lower increase in anxiety in response to a psychosocial stressor than their non-exercising peers. In contrast, a single exercise session does not seem to influence stress-induced changes in interference control and associated DLPFC oxygenation. Thus, such an exercise paradigm may only be valuable in buffering the autonomous stress response.

## Data Availability Statement

The raw data supporting the conclusions of this article will be made available by the authors, without undue reservation.

## Ethics Statement

The studies involving human participants were reviewed and approved by the Ethikkommission Nordwest- und Zentralschweiz, Switzerland. Written informed consent was obtained from all participants. Written informed consent from the participants’ legal guardian/next of kin was not required to participate in this study in accordance with the national legislation and the institutional requirements.

## Author Contributions

MM, SL, and MG were responsible for the conceptualization and study methodology. SL contributed to the Software. MM, SL, and FC contributed to the formal analysis. MM was responsible for the investigation. UP and MG contributed to the resources. MM contributed to the writing of the manuscript (original draft). SL, FC, UP, and MG contributed to the writing of the manuscript (review and editing). UP and MG were responsible for the project supervision. MM was responsible for the project administration. All authors contributed to the article and approved the submitted version.

## Conflict of Interest

The authors declare that the research was conducted in the absence of any commercial or financial relationships that could be construed as a potential conflict of interest.
