# Supplementary material for: The Influence of an Acute Exercise Bout on Adolescents’ Stress Reactivity, Interference Control, and Brain Oxygenation Under Stress
Source: Front Psychol. 2020 Nov 10;11:581965. doi: 10.3389/fpsyg.2020.581965 (PMC7683805; doi:10.3389/fpsyg.2020.581965)
Supplement: Supplementary file 1 [file Data_Sheet_1.docx]

Supplementary material.


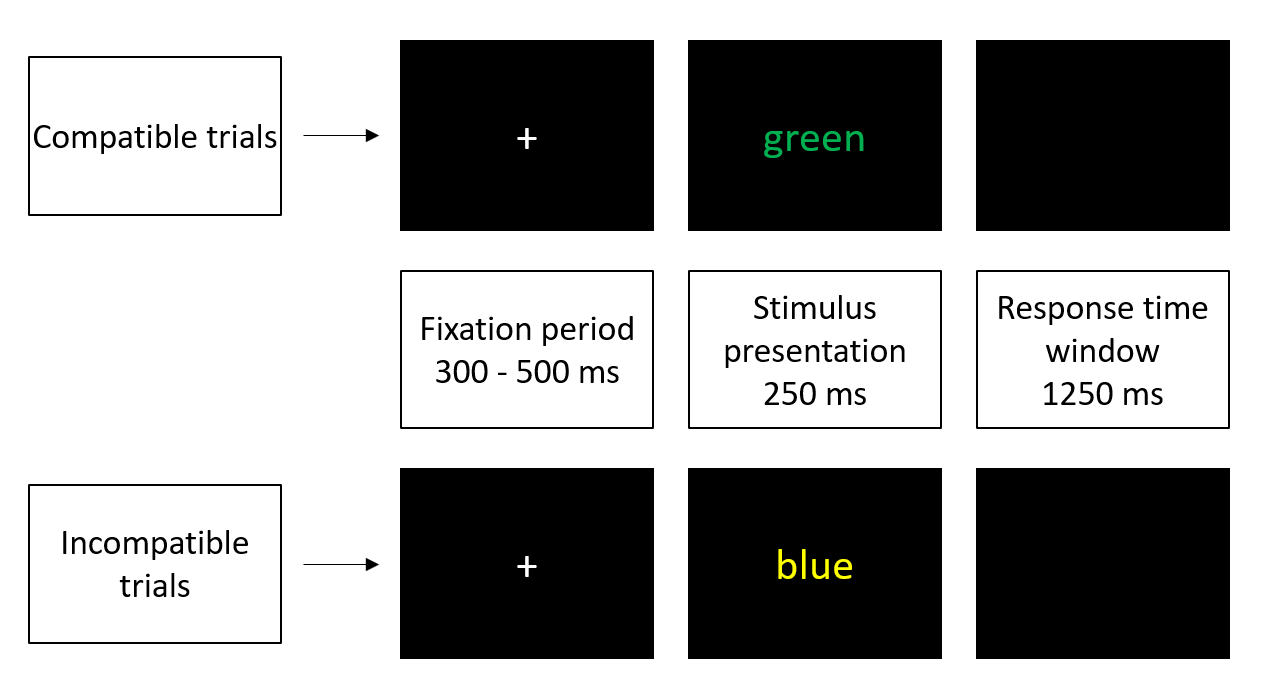


Supplement 1. Examples of Stroop task sequence for compatible and incompatible trials.

*
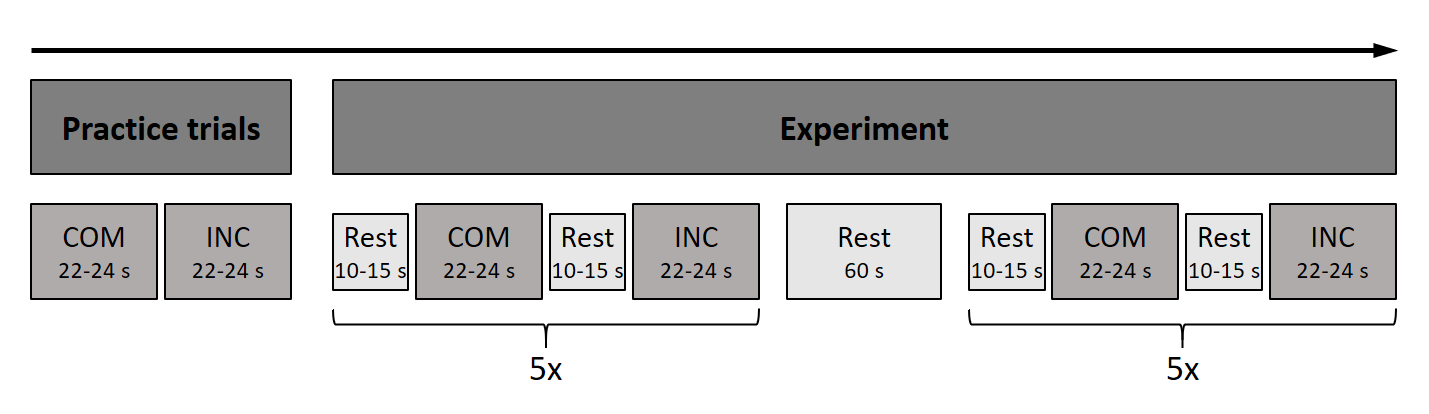
*

Supplement 2. Block design of the Stroop task.

Supplement 3. Average Stroop response accuracy during compatible and incompatible test blocks before and under stress. Error bars are standard errors of the mean (SEM).

Supplement 4. fNIRS channel positions

| Channel | Azimuth | Elevation |
| --- | --- | --- |
| S1_D1 | 91.109 | 129.501 |
| S1_D3 | 80.050 | 141.226 |
| S2_D2 | 89.951 | 50.169 |
| S2_D4 | 81.382 | 59.446 |
| S3_D1 | 81.148 | 120.368 |
| S3_D3 | 69.909 | 132.035 |
| S4_D2 | 79.835 | 38.991 |
| S4_D4 | 70.141 | 47.932 |

S-Source; D-Detector
